# The B-S_2_CALED Score’s Utility in Predicting Stroke Risk in Breast Cancer Patients with Atrial Fibrillation

**DOI:** 10.3390/cancers17223600

**Published:** 2025-11-07

**Authors:** Lakshya Seth, Nickolas Stabellini, Aditya Bhave, Gaurav Gopu, Sandeep Yerraguntla, Ahmed Shetewi, John Lester, Vraj Patel, Stephanie Jiang, Madison James, Stanley Joseph, Sai Kollapaneni, Viraj Shah, Susan Dent, Michael G. Fradley, Lars Køber, Anne Blaes, Avirup Guha

**Affiliations:** 1 Department of Internal Medicine, University of Texas Southwestern Medical Center, Dallas, TX 75390, USA; 2 Department of Medicine, Division of Cardiology, Medical College of Georgia, Augusta University, Augusta, GA 30912, USAjohlester@augusta.edu (J.L.); stjiang@augusta.edu (S.J.);; 3 Department of Hematology-Oncology, University Hospitals Seidman Cancer Center, Cleveland, OH 44106, USA; 4 Wilmot Cancer Institute, Department of Medicine, University of Rochester, Rochester, NY 14624, USA; susan_dent@urmc.rochester.edu; 5 Thalheimer Center for Cardio-Oncology, Division of Cardiology, Department of Medicine, Perelman School of Medicine, University of Pennsylvania, Philadelphia, PA 19104, USA; 6 Department of Cardiology, Rigshospitalet, Copenhagen University Hospital, 2100 Copenhagen, Denmark; lars.koeber.01@regionh.dk; 7 Department of Medicine, Division of Hematology and Oncology, University of Minnesota, Minneapolis, MN 55455, USA; 8 Cardio-Oncology Program, Department of Medicine, Cardiology Division, Medical College of Georgia at Augusta University, Augusta, GA 30912, USA

**Keywords:** breast cancer, arrhythmia, guidelines, risk prediction, screening, thrombosis

## Abstract

**Background:** Breast cancer (BC) patients have heightened risks of atrial fibrillation (AF) and ischemic stroke (IS). Standard IS scores are poorly validated in cancer, omit cancer-specific factors, and guidelines offer no cancer-tailored management. **Objectives:** To develop and validate a novel score to predict IS risk in BC patients with AF. **Methods:** Data sources: UH Seidman Cancer Center (derivation; 40% set aside for internal validation) and MCG Cancer Center (external validation). Adults ≥ 18 years old with DCIS or stage I–IV BC who developed AF after diagnosis were included. Variable selection by LASSO Cox regression; continuous predictors dichotomized via cubic splines; points assigned from multivariable hazards to form B-S_2_CALED. Continuous scores were split into risk groups. Discrimination of categorized B-S_2_CALED versus CHA_2_DS_2_-VASc was assessed with the concordance index (C-index) and net reclassification improvement (NRI). **Results:** In the internal validation cohort (n = 935), 87 patients experienced IS/TIA. The B-S_2_CALED score achieved a C-index of 0.64 (95% CI 0.59–0.70) compared with 0.54 (95% CI: 0.51–0.56) for CHA_2_DS_2_-VASc, yielding a total NRI of 0.188. In the external validation cohort (n = 95), 8 patients developed IS/TIA. The B-S_2_CALED score produced a C-index of 0.77 (95% CI: 0.72–0.83) versus 0.53 (95% CI: 0.51–0.56) for CHA_2_DS_2_-VASc, with a total NRI of 0.563. Similar advantages were observed when the score was treated as a continuous variable. **Conclusions:** The BC-specific B-S_2_CALED score outperformed CHA_2_DS_2_-VASc for predicting thromboembolic events in BC patients with AF. Validation in larger datasets is needed before clinical adoption.

## Simple Summary

Breast cancer patients have a higher risk of atrial fibrillation and ischemic stroke than the general population, and standard ischemic risk scores are poorly validated in cancer patients. This study developed and validated a novel score to predict ischemic stroke risk in breast cancer patients with atrial fibrillation. This breast cancer-specific score outperformed CHA_2_DS_2_-VASc in predicting thromboembolic risk in cancer patients.

## 1. Introduction

Atrial fibrillation (AF) is the most common arrhythmia, affecting ≈59 million people worldwide and carrying a lifetime risk of 1 in 3 after age 45 [1]. Among BC patients, a recent meta-analysis found that the prevalence of AF was 3% [2]. AF increases ischemic stroke (IS) risk five-fold, yet guideline-directed anticoagulation lowers that risk by ≈65% [3]. Cancer further elevates risk: patients have a 63% higher AF incidence [4] and nearly doubles the IS rate [5] versus the general population—likely due to shared risk factors [4], cancer-related physiologic changes [6,7], and antineoplastic cardiotoxicity and thrombogenicity [4,7,8]. One study found that breast cancer survivors under 40 years old had a more than two-fold increased risk of AF, but this risk was not present in older BC survivors, particularly those older than 65 years old [9].

The CHA_2_DS_2_-VASc score, validated for IS prediction in the general AF population, performs poorly in cancer cohorts [10,11] and ignores cancer-specific thromboembolic drivers [7]. In the UK Biobank, its C-index was only 0.57 across cancers and 0.62 in breast cancer (BC) survivors [12]. The 2022 European Society of Cardiology (ESC) guidelines acknowledge the underestimation of risk in oncology patients but offer no alternative [13].

BC is an ideal test case: both the disease and its treatments (anthracyclines, radiation, and endocrine therapy) heighten AF risk [14,15,16,17] and IS [18,19]. New-onset AF after BC diagnosis worsens cardiovascular mortality [15], and BC ranks among the cancers with the highest fatal stroke burden [19].

Given the limited evidence and exclusion of cancer patients from validation studies [20,21], we aimed to create and externally validate a BC-specific score that improves IS/transient ischemic attack (TIA) risk stratification in patients who develop AF after cancer diagnosis. This new score would offer a tailored approach to each patient and incorporate variables that are readily available to clinicians in the electronic medical record to standardize stroke prevention across clinical practice. A BC-specific score would guide clinicians in deciding which patients with AF would benefit the most from initiation of anticoagulation.

## 2. Methods

### 2.1. Inclusion Criteria

We included adults (≥18 yr) with ductal carcinoma in situ (DCIS) or stage I–IV BC who developed AF after diagnosis. Patients lacking diagnosis, death, or last follow-up dates were excluded. Patient records were deidentified, and the study was approved by the Institutional Review Board (IRB).

### 2.2. Outcome

Primary outcome: IS or TIA after AF onset. TIA was included alongside stroke to increase power, given the low event rate in this cancer-specific cohort. While we acknowledge the diagnostic subjectivity of TIA, both outcomes represent cerebral thromboembolic risk and may share overlapping risk profiles, especially in AF populations.

### 2.3. Development

Using the University Hospitals (UH) integrated oncology repository, a large hybrid academic-community tertiary care center in Northeast Ohio, USA, that serves urban, suburban, and rural areas [22], we divided the cohort into derivation (50%), tuning (10%), and internal validation (40%) sets [22,23,24,25]. Eligible patients were diagnosed 2010–2019, ensuring ≥2 yr follow-up (through March 2022). AF, IS, and TIA were identified via ICD codes (Supplemental Table S1).

For the internal validation cohort, we included patients diagnosed with BC between January 2010 and March 2019, providing a follow-up of at least two years through the data collection date (March 2022). Diagnoses of AF, IS, and TIA were captured using ICD codes (Supplemental Table S1). A CONSORT diagram was created to summarize the selection of patients from both cohorts (Supplemental Figure S1).

The covariates encompassed demographics, lifestyle factors, individual-and neighborhood-level social determinants of health (SDOH), cancer characteristics, cancer treatments, comorbidities, medical history, current medications, and laboratory data prior to BC diagnosis (Supplemental Table S2). All variables were collected by medical students (LS, AB, GG, SY, AS, JL, VP, SJ, MJ, SJ, SK), assisted by AG for the first 10 collections and then randomly checked every 5 to 6 charts collected. Medical student abstractors achieved excellent inter-rater reliability (κ = 0.87).

### 2.4. Statistical Analyses

We applied the Least Absolute Shrinkage and Selection Operator (LASSO) Cox regression for variable selection [26]. We used LASSO because it utilizes shrinkage to reduce overfitting and incorporates the joint effect of covariates [27], is not dependent on statistical significance or 95% confidence intervals (CIs), and allows for the inclusion of covariates that may not reach traditional statistical significance thresholds if it seems that the covariates could improve predictive performance [26,28]. This approach is more relevant for risk calculators, where predictors are considered in combination. Variable selection was performed via LASSO with 10-fold cross-validation to determine the optimal lambda. Variables with non-zero coefficients were retained, then transformed into categorical variables using spline-based cutoffs. The selected covariates were determined based on the LASSO cross-validated model’s coefficients at the optimal regularization parameter (lambda) [29]. Continuous predictors were binarized with cubic-spline cut points where HR ≥ 1.00 [30].

After categorization, all covariates were included in a multivariable Cox regression model, from which point values were derived based on the magnitude and precision of the hazard ratios. To transform the final multivariable Cox regression model into an easily usable clinical score similar to CHA_2_DS_2_-VASc, covariates with 95% CI > 1.00 received two points; those whose CI crossed 1.00 received one. The maximum possible score is 10 (Table 1). Cubic spline plots were generated to assess nonlinear relationships between total score and predicted event rates. Inflection points where predicted hazard ratios changed slope informed the threshold for score-based risk categories as follows: 0 = none, 1–4 = low/intermediate, and >4 = high. Score values associated with an HR and 95% CI > 1.00 were classified as high risk, those with an HR and 95% CI crossing 1.00 as low/intermediate risk, and those with an HR and 95% CI < 1.00 as no risk. The no-risk group was defined as such, given the observed risk is lower than the population’s expected risk of IS/TIA, which is higher than seen in our cohort of those with a score of 0 [15].

### 2.5. External Validation

The external validation of the score utilized retrospective data from the Medical College of Georgia (MCG) Cancer Center, an academic tertiary care center that serves much of the urban, suburban, and rural areas in the region. Using manually curated BC records (2010–2022), we identified patients who developed AF post-cancer. Diagnoses and covariates matched the UH variables when available. (Supplemental Table S2).

### 2.6. CHA_2_DS_2_-VASc Calculation

We calculated CHA_2_DS_2_-VASc (maximum = 9) per consensus definitions (Supplemental Table S3) [31,32]. In this population that solely consisted of females, a score of ≤2 was categorized as low/medium risk, while a score of ≥3 was categorized as high risk, based on guidelines that give an Ia class recommendation to initiate anticoagulation in those with a CHA_2_DS_2_-VASc score of ≥2 in men and ≥3 in women [33].

### 2.7. Performance

For both scores, we computed concordance indices (C-index) with 95% CI [34], confusion matrices, and categorical/continuous net reclassification improvement (NRI) [35]. The categorical NRI was computed using the pre-existing risk categories of low/moderate versus high for CHA_2_DS_2_-VASc and none, low-intermediate, and high for the novel score. Since, in addition to the increased thromboembolic risk, BC patients may be at increased risk for bleeding, the intermediate risk category allows for permissive cardiotoxicity [36] by not anticoagulating despite the thromboembolic risk, which could be useful in cancers that increase bleeding risk. The continuous NRI was computed using raw scores. A positive NRI indicated improved predictability of the novel score versus CHA_2_DS_2_-VASc [35]. A Kaplan–Meier curve stratified by risk category assessed whether the model appropriately discriminated between patients at different levels of predicted risk. In a well-calibrated model, the low-risk group would exhibit higher AF-free survival than the medium- and high-risk groups over time. This is seen in our curve, which separates patients by outcome risk, even if no absolute risk values are reported (Supplemental Figure S2). Reporting of this prediction model study adheres to the Transparent Reporting of a Multivariable Prediction Model for Individual Prognosis or Diagnosis (TRIPOD) guidelines [37] (Supplemental Figure S3).

### 2.8. Descriptive Analysis

The data were presented as absolute values and percentages for categorical variables and as mean with standard deviation (SD) or median with interquartile range (IQR) for continuous variables, depending on the data distribution. Categorical variables were compared using Pearson’s χ^2^ test. Data distribution was checked using histograms and the Kolmogorov–Smirnov test, followed by independent samples *t*-tests for normally distributed variables and nonparametric Kruskal–Wallis tests for non-normally distributed variables. The *p*-value of <0.05 was considered significant.

#### Software and Packages

All statistical analyses were performed using R (v4.4.2) [38]. The BiocManager [39], boot [40], prodlim [41], survcomp [42], and survival [43] packages were used.

## 3. Results

### 3.1. Population

Out of the 10,700 total patients in the UH database, 935 were diagnosed with AF after BC diagnosis, with a median follow-up of 2.37 years (IQR 0.64–5.17) after BC diagnosis. The median age at cancer diagnosis was 74 (IQR: 66–80). In terms of cancer treatment, 38.0% of patients underwent surgery, 38.0% of patients received endocrine therapy, 24.0% of patients received chemotherapy, 4.1% received immunotherapy, and 28.2% received radiation therapy (Table 2). In the internal validation cohort, 87 patients had an IS/TIA after AF diagnosis. The significant differences between the 848 patients who did not have an IS/TIA after AF diagnosis and the 87 patients who did have an IS/TIA after AF diagnosis were dyslipidemia (without IS/TIA: 74.5%, with IS/TIA: 86.2%, *p* = 0.02), statin use (without IS/TIA: 53.7%, with IS: 75.9%, *p* < 0.001), antihypertensive use (without IS/TIA: 83.6%, with IS: 93.1%, *p* = 0.02, aspirin use (without IS/TIA: 57.0%, with IS: 79.3%, *p* < 0.001), prior stroke/TIA/embolism (without IS/TIA: 13.7%, with IS: 37.9%, *p* < 0.001), and prior vascular disease (without IS/TIA: 39.9%, with IS: 64.4%, *p* <0.001; Table 2).

Out of the 2274 total patients in the MCG database, 95 patients were diagnosed with AF after BC diagnosis, with a median follow-up of 5.85 years (IQR: 2.74–8.23) after BC diagnosis. The median age at cancer diagnosis was 70 (IQR: 64–74.5). In terms of cancer treatment, 95.79% of patients underwent surgery, 70.3% of patients received endocrine therapy, 25.3% received chemotherapy, 7.4% received immunotherapy, and 61.1% received radiation therapy (Table 2). Eight patients from the validation cohort had an IS/TIA after AF diagnosis. No significant differences in baseline demographics and cardiovascular (CV) events after BC diagnosis were seen between the 87 patients who did not have an IS/TIA after AF diagnosis and the eight patients who did have an IS/TIA after AF diagnosis (Table 2).

### 3.2. Novel Score

The covariates selected from the internal validation cohort by LASSO regression were body mass index, smoking history (current or previous), CKD, antihypertensive use, lipid-lowering therapy (statin use), ethnicity (Black race), diabetes, and stroke/TIA/embolism (prior) (Supplemental Table S4). The acronym B-S_2_CALED is used to capture the individual components of the score. Among these, BMI was categorized as binary using a cutoff of 30. After BMI categorization and based on the updated LASSO model, positive smoking history and prior stroke/TIA/embolism were assigned two points each, while all other components were assigned one point each, resulting in a maximum possible score of 10 (Table 1). Risk categories were defined as 0 = no risk, 1–4 = low/intermediate risk, and >4 = high risk (Supplemental Table S5).

### 3.3. Comparison of the B-S_2_CALED Score with CHA_2_DS_2_-VASc

In the internal validation cohort, the B-S_2_CALED score performed better than CHA_2_DS_2_-VASc in a categorical model (B-S_2_CALED C-index = 0.64 [95% CI: 0.59, 0.70] versus CHA_2_DS_2_-VASc C-index = 0.54 [95% CI: 0.51, 0.56]) and a continuous model (B-S_2_CALED C-index = 0.68 [95% CI: 0.62, 0.73] versus CHA_2_DS_2_-VASc C-index = 0.64 [95% CI: 0.58, 0.70]). Compared to CHA_2_DS_2_-VASc, B-S_2_CALED demonstrated an NRI of 0.188 for the categorical score and 0.150 for the continuous score (Table 3).

In the external validation cohort, the B-S_2_CALED score performed better than CHA_2_DS_2_-VASc in a categorical model (B-S_2_CALED C-index = 0.77 [95% CI: 0.72, 0.83] versus CHA_2_DS_2_-VASc C-index = 0.53 [95% CI: 0.51, 0.56]) and a continuous model (B-S_2_CALED C-index = 0.86 [95% CI: 0.79, 0.94] versus CHA_2_DS_2_-VASc C-index = 0.70 [95% CI: 0.51, 0.89]). Compared to CHA_2_DS_2_-VASc, B-S_2_CALED demonstrated an NRI of 0.563 for the categorical score and 0.695 for the continuous score (Table 3).

## 4. Discussion

This study presents the first externally validated, breast cancer–specific algorithm (B-S_2_CALED) for estimating ischemic stroke and transient-ischemic-attack risk in patients who develop atrial fibrillation after their cancer diagnosis. Across an internal cohort of 935 patients and an external cohort of 95 patients, B-S_2_CALED demonstrated materially higher discrimination and superior net reclassification relative to CHA_2_DS_2_-VASc, the current clinical standard. Additionally, the three different levels of the B-S2CALED score allow for risk stratification of thromboembolic risk by incorporating a middle risk tier, which allows for shared decision-making in high-risk cases. This is in contrast to CHA2DS2-VASc, which would consider initiating anticoagulation with a score as low as one, and often would entail that almost every cancer patient would need anticoagulation. These findings address a critical gap highlighted by recent American Heart Association and European Society of Cardiology guidance, both of which acknowledge inadequate performance of conventional AF risk tools in oncology populations, yet offer no cancer-tailored alternative [13,44].

Both breast cancer and its prevalent therapies—anthracyclines, thoracic radiation, and endocrine manipulation—foster pro-inflammatory, pro-thrombotic, and arrhythmogenic states that may potentiate AF and cerebral embolism [14,15,16,17,18,19]. Traditional scores, designed decades before routine cardio-oncology surveillance, omit these cancer-specific influences as well as key social-determinant and comorbidity profiles that differ markedly between oncology and non-oncology cohorts [7,11,44]. By incorporating chronic kidney disease, obesity, smoking history, and Black race—variables repeatedly associated with heightened stroke incidence and severity [45,46,47,48]—age did not retain independent prognostic weight in our LASSO-derived model. This observation supports the concept that biological aging, accelerated by malignancy and its treatment, may supersede chronological age as a determinant of vascular events in cancer survivors [49,50,51,52]. Future work should explore objective geroscience metrics (e.g., epigenetic clocks, telomere attrition) as candidate variables in cardio-oncology risk stratification.

Prior efforts to refine thromboembolic prediction in cancer patients have either appended a single “cancer” modifier to CHA_2_DS_2_-VASc [53] or tested venous-thromboembolism scores (e.g., Khorana) for arterial events, with limited success [54,55]. The UK Biobank analysis reported modest CHA_2_DS_2_-VASc performance in breast cancer (C-index 0.62) [12]. Our score achieved C-indices of 0.68 (internal) and 0.86 (external), plus positive NRI values in both cohorts, thereby yielding clinically meaningful improvement while retaining simplicity akin to CHA_2_DS_2_-VASc (B-S_2_CALED: max 10 points, three risk tiers).

Suboptimal anticoagulation remains common in cancer patients with AF because clinicians justifiably fear bleeding in a population already exposed to thrombocytopenia, drug–drug interactions, and procedural interventions [56]. A more accurate, oncology-specific risk tool can facilitate nuanced shared decision-making: patients classified as “high risk” by B-S_2_CALED may derive net benefit from anticoagulation despite bleeding concerns, whereas those deemed “low/intermediate” might safely defer therapy, particularly when receiving cardiotoxic regimens or undergoing invasive procedures. Moreover, the score relies on universally available clinical data, avoiding barriers that hamper the implementation of more complex calculators [57], thereby increasing the utility of the score in a clinical setting. Prospective, multicenter validation with adjudicated endpoints and concurrent bleeding assessment is therefore essential before broad adoption.

## 5. Strengths and Limitations

Strengths include the following: (i) a transparent, data-driven variable-selection process; (ii) sizeable internal training and validation subsets; (iii) external validation in an independent health-system cohort with greater Black representation than most cardiology or oncology trials [58,59,60]; and (iv) retention of an intuitive, point-based format conducive to bedside use. Limitations stem from retrospective design; potential misclassification through ICD coding; incomplete treatment documentation in the electronic medical record—especially of anticoagulant exposure—which could impact IS/TIA rates; inclusion of ductal carcinoma in situ; inclusion of TIA diagnosis via ICD coding, which lacks the objectivity of neuroimaging confirmed stroke and may introduce misclassification bias, but this was mitigated by manual checking of charts to ensure clinical correlation with ICD-10 coding; and modest event counts—particularly in the external cohort—that could inflate performance estimates. Additionally, higher medication use in the IS/TIA group may reflect reverse causation—i.e., therapy initiated post-event. This could affect the interpretability of statin and antihypertensive associations, as the timing of medication exposure was not consistently captured.

## 6. Conclusions

B-S_2_CALED score offers a pragmatic, biologically plausible advance over the CHA_2_DS_2_-VASc score for predicting stroke risk in breast cancer patients who develop atrial fibrillation. While confirmation in larger prospective cohorts is required, our findings lay the groundwork for cancer-specific risk stratification paradigms that reconcile thromboembolic prevention with bleeding hazards in an expanding cardio-oncology population.

### Clinical Perspectives

**Competency in Medical Knowledge:** This study emphasizes the importance of derivation of tumor-specific scores for high-incidence cancers with known thromboembolic proclivity (lung, colorectal, prostate).

**Translational Outlook:** Expanding B-S_2_CALED validation to larger, geographically diverse registries will test generalizability and permit recalibration. Integration of circulating biomarkers (e.g., D-dimer, high-sensitivity CRP) and quantitative imaging indices of cardio-oncologic injury may further enhance accuracy without sacrificing usability. Finally, clinical-decision support embedding B-S_2_CALED into electronic health records could standardize stroke-prevention strategies across oncology practices.

## Figures and Tables

**Table 1:** Breakdown of the novel score, the B-S_2_CALED score, by individual components.

B-S_2_CALED Score	
Covariate	Points
BMI > 30	1
Smoking history (current or previous smoker)	2
Stroke/TIA/embolism (prior)	2
CKD	1
Antihypertensive use	1
Lipid-lowering therapy (statin use)	1
Ethnicity (Black race)	1
Diabetes	1
Maximum score	10

**Table 2:** Baseline characteristics and CV events after a breast cancer diagnosis from the internal validation cohort and the external validation cohort were split by whether the patient had an ischemic stroke after an atrial fibrillation diagnosis. * Signifies a statistically significant *p*-value (*p* < 0.05). ^1^ A 2-sided Fisher’s exact test was used to obtain this *p*-value because the χ^2^ assumption was violated due to fewer than 5 expected counts in >1cell. Values are whole numbers with percentages in parentheses unless noted otherwise. ^2^ Selective estrogen receptor modulators (SERM)—Tamoxifen, Raloxifene, Toremifene, and Fulvestrant. Aromatase inhibitors (AI)—Letrozole, Anastrozole, Exemestane, and Testolactone. Chemotherapy—Cyclophosphamide, Doxorubicin, Paclitaxel, Docetaxel, Fluorouracil, Capecitabine, Carboplatin, Methotrexate, Epirubicin, Cisplatin, Vinorelbine, Gemcitabine, Ixabepilone, Eribulin, Mitaxantrone, and Mitomycin.

Variables	Overall Internal Validation Cohort (n = 935)	Patients with IS/TIA After AF in the Internal Validation Cohort (n = 87)	Patients Without IS/TIA After AF in the Internal Validation Cohort (n = 848)	*p*-Value for the Internal Validation Cohort *	Overall External Validation Cohort (n = 95)	Patients with IS/TIA in the External Validation Cohort After AF (n = 8)	Patients Without IS/TIA After AF In the External Validation Cohort (n = 87)	*p*-Value for the External Validation Cohort *
**Median Follow-Up after BC Diagnosis, median (IQR), in years**	2.37 (0.64–5.17)	1.25 (0.39–2.61)	2.54 (0.71–5.56)		5.85 (2.74, 8.23)	6.28 (3.32, 8.60)	5.85 (2.74, 8.22)	
**Age at Diagnosis, median (IQR), in years**	74 (66–80)	74 (68–82)	74 (66–80)	0.17	70 (64–74.5)	70 (66–72)	70 (64–76.5)	0.75
**Race**								
White	724 (77.4)	59 (67.8)	665 (78.4)	0.06	56 (58.95)	2 (25.00)	54 (62.07)	0.06 ^1^
Black	196 (19.9)	24 (27.6)	162 (19.1)		36 (37.89)	6 (75.00)	30 (34.48)	0.05 ^1^
Other	25 (2.7)	4 (4.6)	21 (2.5)		3 (3.16)	0 (0.00)	3 (3.45)	1.00 ^1^
**BMI at Diagnosis, median (IQR)**	31.8 (26.6–38.7)	34 (28.3–41.7)	31.7 (26.6–38.2)	0.05	30 (24–36)	24.2 (23–26.2)	30 (25–36)	0.15
**Smoking History**								
Current	290 (31)	35 (40.2)	255 (30.1)	0.06	11 (11.58)	2 (25.00)	9 (10.34)	0.23 ^1^
**Prior Health Conditions**								
Hypertension	864 (92.4)	84 (96.6)	780 (92)	0.18	72 (75.79)	6 (75.00)	66 (75.86)	1.00 ^1^
Diabetes Mellitus	392 (41.9)	42 (48.3)	350 (41.3)	0.25	34 (35.79)	3 (37.50)	31 (35.63)	1.00 ^1^
Dyslipidemia	707 (75.6)	75 (86.2)	632 (74.5)	0.02 *	37 (38.95)	3 (37.50)	34 (39.08)	1.00 ^1^
Chronic Kidney Disease	328 (35.1)	36 (41.4)	292 (34.4)	0.24	45 (47.37)	5 (62.50)	40 (45.98)	0.47 ^1^
Heart Failure	502 (53.7)	52 (59.8)	450 (53.1)	0.27	4 (4.21)	0 (0.00)	4 (4.60)	1.00 ^1^
Stroke/Transient Ischemic Attack/Embolism	149 (15.9)	33 (37.9)	116 (13.7)	<0.001 *	4 (4.21)	0 (0.00)	4 (4.60)	1.00 ^1^
Depression	303 (32.4)	25 (28.7)	278 (32.8)	0.51	10 (10.53)	0 (0.00)	10 (11.49)	0.59 ^1^
Cardiomyopathy	164 (17.5)	21 (24.1)	143 (16.9)	0.12	-	-	-	-
Cognitive Decline/Dementia	272 (29.1)	26 (29.9)	246 (29)	0.96	-	-	-	-
Anxiety	331 (35.4)	26 (29.9)	305 (36)	0.31	-	-	-	-
Prior vascular disease	394 (42.1)	56 (64.4)	338 (39.9)	<0.001 *	-	-	-	-
**Medication Use**								
Statin Use	521 (55.7)	66 (75.9)	455 (53.7)	<0.001 *	67 (70.53)	8 (100.00)	59 (67.82)	0.10 ^1^
Antihypertensive Use	790 (84.5)	81 (93.1)	709 (83.6)	0.02 *	88 (92.63)	8 (100.00)	80 (91.95)	1.00 ^1^
Metformin Use	104 (11.1)	15 (17.2)	89 (10.5)	0.08	-	-	-	-
Aspirin Use	552 (59)	69 (79.3)	483 (57)	<0.001 *	-	-	-	-
**Cancer Stage**								
III/IV	105 (11.2)	6 (6.9)	99 (11.7)	0.24	7 (7.37)	0 (0.00)	7 (8.05)	1.00 ^1^
**Receptor Status**								
ER+	372 (39.8)	31 (35.6)	341 (40.2)	0.47	75 (78.95)	7 (87.50)	68 (78.16)	1.00 ^1^
PR+	335 (35.8)	32 (36.8)	303 (35.7)	0.93	75 (78.95)	7 (87.50)	68 (78.16)	1.00 ^1^
HER2+	82 (8.8)	9 (10.3)	73 (8.6)	0.72	12 (12.63)	0 (0.00)	12 (13.79)	0.59 ^1^
**Cancer Therapy**								
Endocrine therapy	357 (38.1)	32 (36.8)	325 (38.3)	0.86	67 (70.53)	5 (62.50)	62 (71.26)	0.69 ^1^
SERM use ^2^	120 (12.8)	7 (8.0)	113 (13.3)	0.21	15 (15.79)	1 (12.50)	14 (16.09)	1.00 ^1^
AI use ^2^	365 (39.0)	34 (39.1)	331 (39.0)	1.00	45 (47.37)	5 (62.50)	40 (45.98)	0.47 ^1^
Immunotherapy	38 (4)	3 (3.4)	35 (4.1)	0.98	7 (7.37)	0 (0.00)	7 (8.05)	1.00 ^1^
Chemotherapy ^2^	224 (23.9)	13 (14.9)	211 (24.9)	0.05	24 (25.26)	1 (12.50)	23 (26.44)	0.67 ^1^
Anthracycline use	90 (9.6)	5 (5.7)	85 (10.0)	0.27	8 (8.42)	0 (0.00)	8 (9.20)	1.00 ^1^
Use of HER-2 agents	47 (5.0)	5 (5.7)	42 (5.0)	0.94	11 (11.58)	0 (0.00)	11 (12.64)	0.59 ^1^
Radiation	264 (28.2)	21 (24.1)	243 (28.7)	0.44	58 (61.05)	3 (37.50)	55 (63.22)	0.25
**Surgery**								
Mastectomy	142 (15.2)	12 (13.8)	130 (15.3)	0.82	31 (32.63)	2 (25.00)	29 (33.33)	1.00 ^1^
Lumpectomy	213 (22.8)	19 (21.8)	194 (22.9)	0.93	60 (63.16)	6 (75.00)	54 (62.07)	0.71 ^1^

**Table 3:** Results from the comparison of the B-S_2_CALED score with CHA_2_DS_2_-VASc.

	Internal Validation Cohort		
	B-S_2_CALED C-Index	CHA_2_DS_2_-VASc C-Index	NRI
**Categorical Model**	0.64 [95% CI: 0.59, 0.70]	0.54 [95% CI: 0.51, 0.56]	0.188
**Continuous Model**	0.68 [95% CI: 0.62, 0.73]	0.64 [95% CI: 0.58, 0.70]	0.150
	**External Validation Cohort**		
	**B-S_2_CALED C-index**	**CHA_2_DS_2_-VASc C-index**	**NRI**
**Categorical Model**	0.77 [95% CI: 0.72, 0.83]	0.53 [95% CI: 0.51, 0.56]	0.563
**Continuous Model**	0.86 [95% CI: 0.79, 0.94]	0.70 [95% CI: 0.51, 0.89]	0.695

## Data Availability

University Hospitals Seidman Cancer Center database is available at University Hospitals Cleveland Medical Center and has restricted access to researchers with IRB approval. The Medical College of Georgia Cancer Center database is available at the Medical College of Georgia and has access restricted to users with IRB approval.

## Supplementary Materials

The following supporting information can be downloaded at: https://www.mdpi.com/article/10.3390/cancers17223600/s1; Table S1: ICD codes for diagnoses included in the internal validation cohort; Table S2: Covariates initially available in both the internal validation cohort and the external validation cohort during development; Table S3: Breakdown of the CHA2DS2-VASc score by individual component. *Prior myocardial infarction, aortic plaque, and peripheral arterial disease; Table S4: Covariates selected from the internal validation cohort by LASSO regression, categorized by multivariable Cox regression, and categorized by risk category. *Signifies a statistically significant *p* value (*p* < 0.05); Table S5: Risk categories for the novel score; Figure S1: Kaplan–Meier curve stratified by risk category; Figure S2: Transparent reporting of a multivariable prediction model for individual prognosis or diagnosis (TRIPOD) checklist. Figure S3. Transparent reporting of a multivariable prediction model for individual prognosis or diagnosis (TRIPOD) checklist.

## Abbreviations

AF = atrial fibrillation, IS = ischemic stroke, BC = breast cancer, TIA = transient ischemic attack, DCIS = ductal carcinoma in situ, SDOH = social determinants of health, LASSO = Least Absolute Shrinkage and Selection Operator, NRI = net reclassification improvement.
