# Supplementary material for: The B-S2CALED Score’s Utility in Predicting Stroke Risk in Breast Cancer Patients with Atrial Fibrillation
Source: Cancers (Basel). 2025 Nov 7;17(22):3600. doi: 10.3390/cancers17223600 (PMC12651892; doi:10.3390/cancers17223600)
Supplement: Supplementary file 1 [file cancers-17-03600-s001.zip › cancers-3920106-supplementary.pdf]

The B-S$_2$CALED Score's Utility in Predicting Stroke Risk in Breast Cancer Patients with Atrial Fibrillation

Table S1. ICD codes for diagnoses included in the internal validation cohort. Page 2.
Table S2. Covariates initially available in both the internal validation cohort and the external validation cohort during development. Pages 3-8.
Table S3. Breakdown of the CHA$_2$DS$_2$-VASc score by individual component. *Prior myocardial infarction, aortic plaque and peripheral arterial disease. Page 9.
Table S4. Covariates selected from the internal validation cohort by LASSO regression, categorized by multivariable cox regression, and categorized by risk category. *Signifies a statistically significant p value ($p < 0.05$). Page 10.
Table S5. Risk categories for the novel score. Page 11.
Figure S1. CONSORT Diagram. Page 12.
Figure S2. Kaplan-Meier curve stratified by risk category. Page 13.
Figure S3. Transparent Reporting of a Multivariable Prediction Model for Individual Prognosis or Diagnosis (TRIPOD) Checklist. Page 14.

Table S1. ICD codes for diagnoses included in the internal validation cohort.

Outcome/Comorbidity	ICD-9 and ICD-10 codes
Atrial Fibrillation	427.31; I48.X
Ischemic Stroke/Transient Ischemic Attack (TIA)	433.01; 433.11; 433.21; 433.31; 433.81; 433.91; 434.01; 434.11; I63.XX; 435.XX; G45.XX
Cardiomyopathy	425; I42.XX
Hypertension	401.1; 401.9; I10.XX; I11.XX; I12.XX; I13.XX; I14.XX; I15.XX
Chronic Kidney Disease	585.XX, N18.XX
Dyslipidemia	272.XX; E78.XX
Obesity	278.XX; E78.XX
Diabetes	249.XX; 250.XX; E08.XX; E09.XX; E10.XX; E11.XX; E13.XX; E14.XX
Cognitive Decline/Dementia	331.48; 294.20; 290.10; 290.11; 290.13; 290.21; 290.8; 799.52; 780.91; 294.9; 799.5; G31.84; R41.XX; F03.90
Depression	311.XX; 296.82; 296.33; 296.35; 296.30; F32.89; F33.XX; F32.9
Anxiety	300.00; 300.4; 300.02; F41.9; F41.8; F41.1
Bipolar Disorder	296.80; 296.50; 296.89; 296.7; F31.XX
Prior Congestive Heart Failure	428.XX; I50.XX
Prior Stroke/TIA/Embolism	433.01; 433.11; 433.21; 433.31; 433.81; 433.91; 434.01; 434.11; 433.91; 434.XX; 435.XX; 436.XX; 444.XX; I63.XX; I64.XX; G45.XX; I74.XX
Prior Vascular Disease	410.XX; 412.XX; 440.XX; 441.XX; 442.9; 443.XX; I21.XX; I25.2; I70.XX; I71.XX; I72.XX; I73.XX

Table S2. Covariates initially available in both the internal validation cohort and the external validation cohort during development.

Cohort	Covariate	Original Categories	Recategorization

**Internal validation cohort**	**Gender**	Male	Male
		Female	Female
	**Age at diagnosis**	Continuous	
	**Race**	"White", "White~White"	White
		"Black", "Black or african american", "Black~black"	Black
		Others, NA	Other/unknown
	**Ethnicity**	"Mexican, Mexican American, or Chicano/a", "Mexican", "Mexican (includes Chicano)", "Central American", "Central Amer", "CENTRAL AMER", "Latin American", "Hispanic", "HISPANIC", "Spanish, NOS; Hispanic, NOS; Latino; NOS", "Other Spanish/Hispanic origin(included European)", "South American", "Puerto Rican", "PUERTO RICAN", "Hispanic or Latino/Spanish", "Spaniard", "SPANIARD"	Hispanic
		Non spanish; Non Hispanice", "Non-Hispanic", "Not Hispanic, Latino/a, or Spanish origin", "Non-Hispanic", "Not Hispanic", "NOT HISPANIC", "Not Hispanic or Latino"	Non-hispanic
		NA, "Patient Declined", "Pt Not Available", "Pt Declined","PT DECLINED", "PT NA", "-", "Other"	Other/unknown
	**Date of death**	Date	
	**Smoking status**	Yes	Current
		Former	Previous
		No	Never
	**Diagnosis date**	Date	
	**Vital status**	Death	Death
		Alive	Alive
	**Last follow-up date**	Date	
	**Annual Income**	Continuous	
	**Number of household members**	Continuous	
	**Distance to closest relatives**	-1	Unknown
		0	<25 miles
		1	>25 miles
	**Household annual income**	-1	Unknown
		1	<= 25,000
		2,3,4,5	25,001-50,000
		6,7,8	50,001-100,000
		9,10,11	>100,000
	**Number of transportation properties owned**	Continuous	
	**Married**	"Married", "Not married"	Binary (yes vs no)
	**Owns current residence**	"Owner", "Tenant"	Binary (yes vs no)
	**IS/TIA After Afib**	Captured according to ICD codes	Binary (yes vs no)

**Mastectomy**	Binary (yes vs no)	
**Lumpectomy**	Binary (yes vs no)	
**Endocrine therapy**	Binary (yes vs no)	
**Radiotherapy**	Binary (yes vs no)	
**Stage**	"I", "II"	Not advanced stage
	"III", "IV"	Advanced stage
**Cardiomyopathy**	Captured according to ICD codes	Binary (yes vs no)
**Hypertension**	Captured according to ICD codes	Binary (yes vs no)
**Chronic Kidney disease**	Captured according to ICD codes	Binary (yes vs no)
**Dyslipidemia**	Captured according to ICD codes	Binary (yes vs no)
**Obesity**	Captured according to ICD codes	Binary (yes vs no)
**Diabetes**	Captured according to ICD codes	Binary (yes vs no)
**HER2**	"Positive"	Positive
	"Negative", "Indeterminate"	Not positive
**ER**	"Positive"	Positive
	"Negative", "Indeterminate"	Not positive
**PR**	"Positive"	Positive
	"Negative", "Indeterminate"	Not positive
**Oncotype Score**	Continuous	
**Cognitive decline/dementia**	Captured according to ICD codes	Binary (yes vs no)
**Depression**	Captured according to ICD codes	Binary (yes vs no)
**Anxiety**	Captured according to ICD codes	Binary (yes vs no)
**Bipolar disorder**	Captured according to ICD codes	Binary (yes vs no)
**Metformin**	"Metformin", "Fortamet", "Glucophage", "Glumetza", "Riomet"	Binary (yes vs no)
**Statin**	Drugs ending with "statin", except "nystatin"	Binary (yes vs no)
**Antihypertensive medication**	"Lisinopril", "losartan", "amlodipine", "metoprolol", "hydrochlorothiazide", "enalapril", "ramipril", "candersartan", "valsartan", "diltiazem", "nifedipine", "clonidine", "propranolol", "carvedilol"	Binary (yes vs no)
**Aspirin**	"Aspirin"	Binary (yes vs no)
**SLGT2**	"Canagliflozin", "Dapagliflozin", "Empagliflozin", "Ertugliflozin", "Sotagliflozin"	Binary (yes vs no)
**GLP1**	"Liraglutide", "Exenatide", "Dulaglutide", "Semaglutide", "Lixisenatide", "Tirzepatide"	Binary (yes vs no)
**BMI**	Continuous	
**Prior Congestive Heart Failure**	Captured according to ICD codes	Binary (yes vs no)
**Prior Stroke/TIA/Embolism**	Captured according to ICD codes	Binary (yes vs no)

	**Prior vascular disease**	Captured according to ICD codes	Binary (yes vs no)
	**Anthracycline**	"Daunorubicin", "Doxorubicin", "Epirubicin", "Idarubicin", "Mitoxantrone", "Valrubicin", "Cerubidine", "Daunoxome", "Adryamycin", "Doxil", "Lipodox", "Ellence", "Idamycin", "Novantrone", "Valstar"	Binary (yes vs no)
	**Taxane**	"Paclitaxel", "Doxetaxel", "Taxotere", "Taxol", "Docefrez"	Binary (yes vs no)
	**HER2 Agent**	"Trastuzumab", "Pertuzumab", "Trastuzumab-ematansine", "Lapatinib", "Neratinib", "Tucatinib", "Herceptin", "Perjeta", "Kadcyla", "Tykerb", "Nerlynx", "Tukysa"	Binary (yes vs no)
	**CKD 4/6 Inhibitors**	"Ribociclib", "Palbociclib", "Abemaciclib", "Kisqali", "Ibrance", "Verzenios"	Binary (yes vs no)
	**Cyclophosphamide**	"Cyclophosphamide", "Cytoxan"	Binary (yes vs no)
	**Leuprolide**	"Lupron", "Leuprolide"	Binary (yes vs no)
	**SERM use**	“Tamoxifen”, “Raloxifene”, “Toremifene”, “Fulvestrant”	Binary (yes vs no)
	**AI use**	“Letrozole”, “Anastrozole”, “Exemestane”, “Testolactone”	Binary (yes vs no)
	**Creatinine**	Continuous	
	**Egfr**	Continuous	
	**Hemoglobin**	Continuous	
	**Platelets**	Continuous	
	**Time to event**	Obtaining subtracting event date - AF/diagnosis date	
**External Validation Cohort**			
	**Race**	"White"	White
		"Black"	Black
		"Asian"	Asian
		"Hispanic"	Hispanic
		"Other"	Other
	**Date of birth**	Date	
	**Zip code**	Zip Code	
	**Age at diagnosis**	Continuous	
	**Number of household members**	Continuous	
	**Smoking status**	"Current"	Current
		"Former"	Former
		"No"	No
	**Past medical history** (hypertension, diabetes, dyslipidemia, heart failure, atrial fibrillation, atrial flutter, myocardial infarction, ischemic stroke/transient ischemic attack, chronic kidney disease, coronary artery disease, peripheral artery disease, chronic obstructive pulmonary disease, rheumatological disease, dementia, hypothyroidism, depression)	Captured through manual screening	Binary (yes vs no)
	**Medication use** (statins, antihypertensives, ezetimibe, PCSK9 inhibitors, bempedoic acid, fibrates,	Captured through manual screening	Binary (yes vs no)

omega-3/fish oil, insulin, metformin, antidiabetic)		
**Cancer treatments** (chemotherapy/endocrine/immunotherapy use, use of radiation therapy, surgical management)	Aggretated from IT department	Binary (yes vs no)
**SERM use**	"Tamoxifen", "Raloxifene", "Toremifene", "Fulvestrant"	Binary (yes vs no)
**AI use**	"Letrozole", "Anastrozole", "Exemestane", "Testolactone"	Binary (yes vs no)
**Laterality**	"Left"	Left
	"Right"	Right
**HER2**	"Positive"	Positive
	"Negative", "Indeterminate"	Not positive
**ER**	"Positive"	Positive
	"Negative", "Indeterminate"	Not positive
**PR**	"Positive"	Positive
	"Negative", "Indeterminate"	Not positive
**Laboratory data** (microalbumin, creatinine, eGFR, LDL, HDL, triglycerides)	Continuous	
**Vital signs** (systolic blood pressure, diastolic blood pressure)	Continuous	

Table S3. Breakdown of the CHA_2DS_2-VASc score by individual component. *Prior myocardial infarction, aortic plaque and peripheral arterial disease.

Letter	Risk Factor	Points
C	Congestive heart failure	1
H	Hypertension	1
A_2	Age ≥ 75	2
D	Diabetes	1
S_2	IS/TIA/thromboembolism	2
V	Vascular disease*	1
A	Age 65-74	1
Sc	Female sex	1
	Maximum score	9

Table S4. Covariates selected from the internal validation cohort by LASSO regression, categorized by multivariable cox regression, and categorized by risk category. *Signifies a statistically significant p value ($p < 0.05$).

	LASSO Regression	
	**HR [95% CI]**	**p-value***
CKD	1.01 [0.62, 1.64]	0.96
Antihypertensive	1.32 [0.59, 2.97]	0.50
Smoking history	1.55 [1.01, 2.37]	0.04*
Black race	1.30 [0.80, 2.11]	0.29*
Statin	1.55 [0.91, 2.64]	0.10
Diabetes	0.99 [0.62, 1.59]	0.97
BMI	1.00 [0.98, 1.02]	0.94
Prior Stroke/TIA/Embolism	3.17 [2.02, 4.98]	< 0.001*

Multivariable COX Regression		
	**HR [95% CI]**	**p-value**
CKD	1.00 [0.62, 1.62]	0.99
Antihypertensive	1.33 [0.59, 2.97]	0.49
Smoking history	1.53 [1.00, 2.35]	0.04*
Black race	1.30 [0.80, 2.10]	0.29
Statin	1.52 [0.89, 2.58]	0.12
Diabetes	0.94 [0.59, 1.52]	0.84
BMI	1.26 [0.82, 1.94]	0.30
Prior Stroke/TIA/Embolism	3.26 [2.07, 5.13]	< 0.001*
**Categorized by Risk Category**		
	**HR [95% CI]**	**p-value**
Intermediate risk	1.70 [0.41, 7.04]	0.47
High risk	4.65[1.13, 19.12]	0.03*

Table S5. Risk categories for the novel score.

Risk Categories	
**Points**	**Category**
0	No risk
0-4	Low/Intermediate risk
>4	High risk

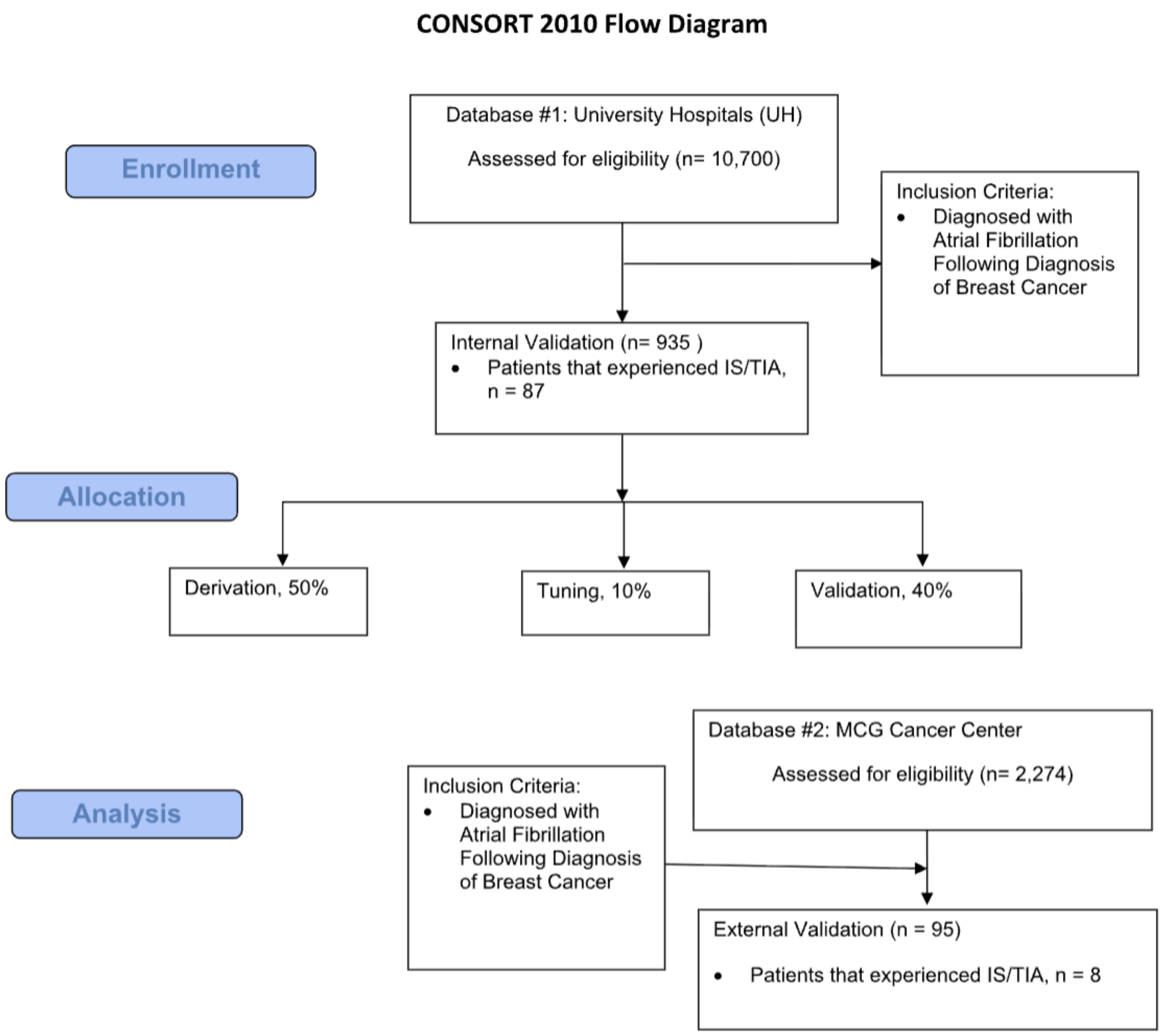

Figure S1. CONSORT Diagram.

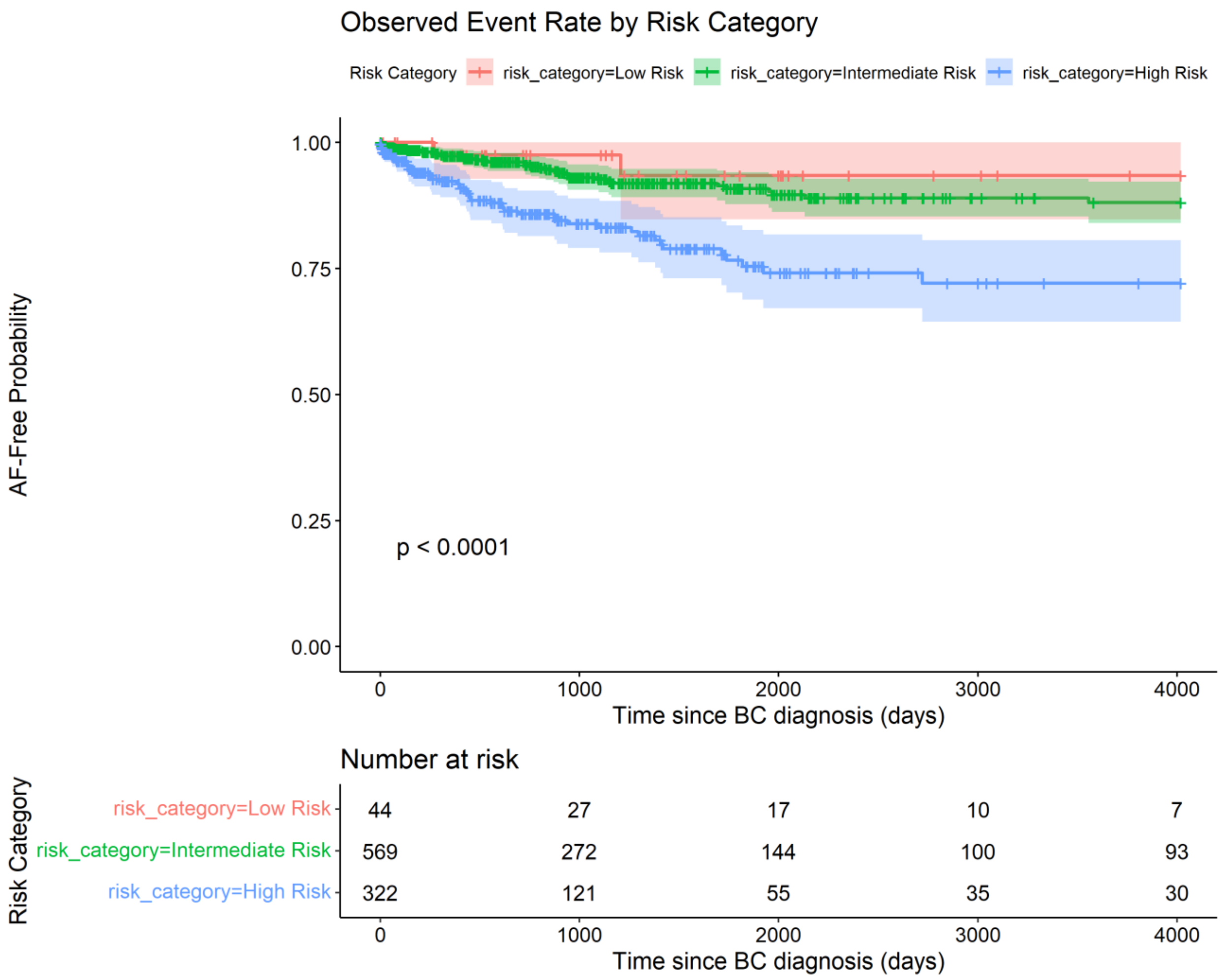

Figure S2. Kaplan-Meier curve stratified by risk category.

TRIPOD

TRIPOD Checklist: Prediction Model Development and Validation

Section/Topic	Item		Checklist Item	Page
**Title and abstract**				
Title	1	D;V	Identify the study as developing and/or validating a multivariable prediction model, the target population, and the outcome to be predicted.	1
Abstract	2	D;V	Provide a summary of objectives, study design, setting, participants, sample size, predictors, outcome, statistical analysis, results, and conclusions.	3
**Introduction**				
Background and objectives	3a	D;V	Explain the medical context (including whether diagnostic or prognostic) and rationale for developing or validating the multivariable prediction model, including references to existing models.	2-3
	3b	D;V	Specify the objectives, including whether the study describes the development or validation of the model or both.	2-3
**Methods**				
Source of data	4a	D;V	Describe the study design or source of data (e.g., randomized trial, cohort, or registry data), separately for the development and validation data sets, if applicable.	5
	4b	D;V	Specify the key study dates, including start of accrual; end of accrual; and, if applicable, end of follow-up.	5-6
Participants	5a	D;V	Specify key elements of the study setting (e.g., primary care, secondary care, general population) including number and location of centres.	5-6
	5b	D;V	Describe eligibility criteria for participants.	5
	5c	D;V	Give details of treatments received, if relevant.	-
Outcome	6a	D;V	Clearly define the outcome that is predicted by the prediction model, including how and when assessed.	7-8
	6b	D;V	Report any actions to blind assessment of the outcome to be predicted.	-
Predictors	7a	D;V	Clearly define all predictors used in developing or validating the multivariable prediction model, including how and when they were measured.	23
	7b	D;V	Report any actions to blind assessment of predictors for the outcome and other predictors.	-
Sample size	8	D;V	Explain how the study size was arrived at.	5
Missing data	9	D;V	Describe how missing data were handled (e.g., complete-case analysis, single imputation, multiple imputation) with details of any imputation method.	-
Statistical analysis methods	10a	D	Describe how predictors were handled in the analyses.	6-7
	10b	D	Specify type of model, all model-building procedures (including any predictor selection), and method for internal validation.	5-7
	10c	V	For validation, describe how the predictions were calculated.	5-7
	10d	D;V	Specify all measures used to assess model performance and, if relevant, to compare multiple models.	8
	10e	V	Describe any model updating (e.g., recalibration) arising from the validation, if done.	8
Risk groups	11	D;V	Provide details on how risk groups were created, if done.	7
Development vs. validation	12	V	For validation, identify any differences from the development data in setting, eligibility criteria, outcome, and predictors.	-
**Results**				
Participants	13a	D;V	Describe the flow of participants through the study, including the number of participants with and without the outcome and, if applicable, a summary of the follow-up time. A diagram may be helpful.	9-10, 25
	13b	D;V	Describe the characteristics of the participants (basic demographics, clinical features, available predictors), including the number of participants with missing data for predictors and outcome.	9-10, 25-28
	13c	V	For validation, show a comparison with the development data of the distribution of important variables (demographics, predictors and outcome).	25-28
Model development	14a	D	Specify the number of participants and outcome events in each analysis.	9-10, 25
	14b	D	If done, report the unadjusted association between each candidate predictor and outcome.	-
Model specification	15a	D	Present the full prediction model to allow predictions for individuals (i.e., all regression coefficients, and model intercept or baseline survival at a given time point).	Supplemental 10
	15b	D	Explain how to the use the prediction model.	10
Model performance	16	D;V	Report performance measures (with CIs) for the prediction model.	10-11
Model-updating	17	V	If done, report the results from any model updating (i.e., model specification, model performance).	-
**Discussion**				
Limitations	18	D;V	Discuss any limitations of the study (such as nonrepresentative sample, few events per predictor, missing data).	13
Interpretation	19a	V	For validation, discuss the results with reference to performance in the development data, and any other validation data.	10-11
	19b	D;V	Give an overall interpretation of the results, considering objectives, limitations, results from similar studies, and other relevant evidence.	11-12
Implications	20	D;V	Discuss the potential clinical use of the model and implications for future research.	13-14
**Other information**				
Supplementary information	21	D;V	Provide information about the availability of supplementary resources, such as study protocol, Web calculator, and data sets.	-
Funding	22	D;V	Give the source of funding and the role of the funders for the present study.	-

Figure S3. Transparent Reporting of a Multivariable Prediction Model for Individual Prognosis or Diagnosis (TRIPOD) Checklist.
